# miRNA 933 Expression by Endothelial Cells is Increased by 27-Hydroxycholesterol and is More Prevalent in Plasma from Dementia Patients

**DOI:** 10.3233/JAD-180201

**Published:** 2018-07-03

**Authors:** Irundika H.K. Dias, Caroline L. Brown, Kiran Shabir, M. Cristina Polidori, Helen R. Griffiths

**Affiliations:** a Aston Medical Research Institute, Aston Medical School, Aston University, Birmingham, UK; b Life and Health Sciences and Aston Research Centre for Healthy Ageing, Aston University, Birmingham, UK; c Institute of Biochemistry and Molecular Biology I, Heinrich-Heine-University, Dusseldorf, Germany; d Ageing Clinical Research, Department Medicine II, University Hospital of Cologne, Cologne, Germany; e Faculty of Health and Medical Sciences, University of Surrey, Stag Hill, Guildford, UK

**Keywords:** Alzheimer’s disease, dementia, glutathione, inflammation, miRNA, neurotrophic factor, oxysterol, redox, vascular

## Abstract

Alzheimer’s disease (AD) etiology is complex; gene and environmental risk factors may interact to predispose to disease. From single nucleotide polymorphism analyses and genome-wide association studies, a number of candidate risk genes for the onset of AD have been identified and cluster around lipid metabolism and inflammation. We hypothesized that endothelial cells which line the blood-brain barrier are likely to be critical mediators of systemic metabolism within the brain. Therefore, we have studied the effect of 27 hydroxycholesterol (27-OHC) on microvascular endothelial cell (HMVEC) redox state, inflammatory cytokine secretion, and microRNA (miR) expression. Using a transwell method, we have studied directional secretion profiles for the proinflammatory cytokines TNF*α* and IL-6 and confirmed that 27-OHC induces discrete and directional inflammatory molecular signatures from HMVEC. The lipids caused depletion of cellular glutathione and cytokine secretion is HMVEC-redox state-dependent. Discovery miR expression change in HMVEC with and without 27-OHC treatment was undertaken. We selected three genes for further analysis by qPCR; miR-144 and 146 expression, which are anti-inflammatory and redox regulating modulators, were not affected significantly by 27-OHC. However, increased expression of a putative neurotrophic regulatory factor miR933 in HMVEC with 27-OHC was confirmed by qPCR. In plasma from patients with dementia, all three miR were found at significantly elevated levels compared to healthy older adults. These data highlight that 27-OHC has an important regulatory effect on endothelial microvascular cells to increase expression of a miR (–933) and secretion of inflammatory cytokines that are elevated in plasma from dementia patients.

## INTRODUCTION

A number of comorbidities have been associated with increased risk of developing Alzheimer’s disease (AD) including metabolic syndrome, obesity, type 2 diabetes, and chronic inflammatory disease [1]. In partial support of the theory that inflammation plays a significant role in AD, some but not all observational studies have suggested that an inverse association exists between use of non-steroidal anti-inflammatory drug (NSAID) use and the risk of AD [2–6]. Similarly, evidence from observational studies has grown for an association between elevated cholesterol, the ApoE4 allele, and AD [7].

Metabolic and hyperlipidemic conditions exert oxidative and inflammatory stress on endothelial cells that line blood vessels. Under stress conditions, progenitor endothelial cells shed microvesicles which are loaded with paracrine signals including microRNA (miR). miR have emerged as critical regulators of gene expression acting at the post-transcriptional level to either promote degeneration or inhibit translation of target mRNA [8]. A number of studies have investigated miR in postmortem brains from AD patients [9, 10]. miR are also found in extracellular fluids where they may exert paracrine effects on neighboring cells. There are several potential explanations for their presence in plasma including apoptosis, shedding of extracellular vesicles (EVs) and active export by the Argonaute family from inflammatory and vascular cells [11].

“NeurimmR”, a class of miR regulating both neuronal and immune functions, have been described [12]. Within this class, plasma miR-132 and miR-134 families have been reported as biomarkers of mild cognitive impairment [13].

The blood-brain barrier (BBB) segregates peripheral from central lipid metabolism in health. BBB dysfunction is associated with the accumulation of several vasculotoxic and neurotoxic molecules within the brain parenchyma, a reduction in cerebral blood flow, and hypoxia. Endothelial cells are a major source of plasma miR in systemic vascular disease. Increased shedding of EVs containing miR-132 and 134 family members has been described in hypercholesterolemia [13]. Our understanding of roles for these molecules in crosstalk from the body to the brain is still in its infancy; they may be mediators of signaling across the BBB in AD.

Hypercholesterolemia is associated with BBB damage, leakage and inflammation in the cortex of animals [14]. Together, these vascular-derived insults have been suggested to initiate and/or contribute to neuronal degeneration [15]. One class of lipid molecules with neurotoxic properties are the hydroxycholesterols [16]. 27-hydroxycholesterol (27-OHC), which is produced systemically, is pro-oxidant and increases production of amyloid-*β* (A*β*) by neuronal cells in culture [17].

We have previously shown that the lipid fraction of LDL is responsible for microvascular endothelial cell barrier leakage due to disruption of tight junctions, can increase secretion of inflammatory mediators such as TNF-*α* and that *in vitro*-oxidized LDL is more inflammatory toward endothelial cells than native LDL [18]. Moreover, lipids extracted from the LDL of patients with AD or hypercholesterolemia are more inflammatory to microvascular endothelial cells than lipids from the LDL of healthy subjects [18].

We and others have described an increase in systemic oxidized lipids in patients with AD and AD with vascular complications [18–21]. Whether oxidized lipids can modulate endothelial cell miR expression remains unknown. Therefore, the objectives of this study are to explore the sensitivity of miR expression by microvascular endothelial cells to the cholesterol oxidation product 27-OHC *in vitro* and relate this to miR expression in plasma from elderly people with and without AD. Our aim is to identify specific oxysterol-sensitive circulating miR with relevance for AD at the BBB.

## MATERIALS AND METHODS

### Microvascular endothelial model

Human microvascular endothelial cells (HMVEC) were maintained as previously described [18], then seeded (3×10^5^/ml) onto 24-well polycarbonate inserts and cultured for 7 days. 27-OHC (0–25*μ*M) was added to the apical surface of the endothelial barrier for 24 h.

#### Cell viability

Cell viability was measured using the Cell Titer-Blue^®^ (Promega, UK) viability assay. Transwells with cultured cells were incubated with 100*μ*l of 1:10 diluted Cell Titer-Blue^®^ reagent for 4 h, supernatants from Transwell^®^ inserts were removed to a 96 well plate, and the fluorescence was measured after excitation at 560 nm and emission at 590 nm using a Spectra Max Gemini XS fluorimeter (Molecular Devices, UK).

#### Reduced glutathione (GSH) analysis and cytokine secretion

HMVEC reduced GSH levels were measured by GSH-Glo assay^®^ (Promega, UK). Following the cell treatments with lipids, cell culture media was collected and cells were pelleted by centrifugation (200 *g*, 10 min). Cell free media containing secreted cytokines was stored at – 20°C until analysis for interleukin (IL)-6 and tumor necrosis factor (TNF)*α* by ELISA (Peprotech, UK).

#### Barrier permeability

Change in endothelial barrier permeability post 27-OHC treatment was analyzed by addition of 200*μ*l of 1 mg/ml FITC-dextran apically for 24 h. The basolateral and apical media was collected, the apical surface washed twice, then the washes were combined with the apical media collected. Fluorescence of the media was read at the following wavelengths; excitation 488 nm and emission wavelength 520 nm using a Spectra Max Gemini XS fluorimeter (Molecular Devices). The percentage fluorescence in the basolateral compartment was calculated as a measure of cell permeability.

#### miR isolation

Endothelial cells were harvested and washed in PBS, then the cell pellet was lysed in 700*μ*l of Trizol reagent, vortexed and centrifuged at 10,000 *g* for 10 min at 4°C. The supernatants were carefully transferred to fresh, RNase free Eppendorfs and stored at – 80°C until extraction. For extraction, 140*μ*l of chloroform was added, the tube sealed and shaken vigorously for approximately 30 s. The mixture was centrifuged at 12,000× *g* 4°C for 15 min in order to separate the mixture into an upper aqueous phase and a lower organic phase. The upper aqueous phase was collected (approximately 350*μ*l) and mixed with 1.5×volume of 100% ethanol (approximately 525*μ*l) by pipetting.

#### Clinical populations

Healthy subjects (*n* = 10) and patients with dementia, with and without vascular complications (*n* = 10 in each group) were recruited from the Unit of Cognitive Frailty, Neurology Outpatient Clinic, Cologne, Germany. Diagnosis of AD was by using NINCDS–ADRDA criteria either in the presence of vascular comorbidities and risk factors (elevated intima-media thickness of the common carotid artery and/or type 2 diabetes mellitus) (AD-Plus group) or without cardiovascular comorbidities and risk factors (AD group) (Table 1) [22]. Informed consent was obtained from the patients or their care givers according to severity of disease and the study was approved by the local ethics committee. All procedures on human subjects were done in accord with the Helsinki Declaration of 1975. A single blood sample was taken and plasma was prepared by centrifugation (200 *g*, 30 min).

For extraction of miR, following the manufacturer’s instructions, 1 ml of Qiazol lysis reagent was added to plasma samples (200*μ*l) which were then vortexed and incubated for 5 min at room temperature. All plasma samples were spiked with an internal standard (1.6×10^8^ copies/*μ*l of C.*elegans* miR-39). Chloroform (200*μ*l) was added, then samples were vortexed and incubated for 3 min at room temperature. The samples were centrifuged for 15 min at 12000× *g* at 4°C. The upper aqueous phase was collected from each sample into a new collection tube and 900*μ*l of 100% ethanol was added.

**Table 1:** Demographics of healthy control and patients with AD or AD plus

	Control (*n* = 10)	AD (*n* = 10)	AD-Plus (*n* = 10)	*p*
Age (y)	75±2.7	81.2±2.5	79.3±1.5	NS
BMI Kg/m^2^	23.8±0.32	23.9±0.62	26.1±0.31	*p* < 0.05 Control versus AD-plus
% of ApoE4 carriers	10%	33%	33%	
Cognitive function scores (MMSE and clock test)	29.5±0.2	20.2±2.0	19.8±2.3	*p* < 0.0001 Compared to control
	16±0.4	7.0±1.5	7.2±1.3

### miRNA extraction from plasma

For miRNA extraction, the miRNeasy serum/plasma kit (Qiagen; Skelton House, Lloyd Street North, Manchester M15 6SH) was used. The samples were added to RNeasy MinElute spin columns and centrifuged for 15 s at 8000× *g*. After washing the spin columns with RWT buffer once followed by twice with Buffer RPE and once with 80% ethanol. RNase-free water (14*μ*l) was added to elute the RNA and columns were centrifuged for 1 min at full speed. miRNA concentration was determined by NanoDroptrademark (Thermofisher Scientific, UK).

### Microarray and GeneSpring^®^ analysis

miRNA quantity was validated using an Agilent® 2100 Bioanalyzertrademark instrument (Agilent Technologies LDA UK Ltd, Stockport, UK) at the University of Birmingham. A single discovery microarray experiment was performed using miR extracted from endothelial cells after oxidized lipid treatment in duplicate according with the manufacturer’s instructions. Fluorescent array images were collected using Agilent DNA microarray scanner and microarray data was analyzed using GeneSpring GX software (Agilent Technologies LDA UK Ltd, Stockport, UK). Shift to 75.0 percentile was used as normalization and median of all samples was applied as baseline transformation. Up- and downregulated genes were explored versus an experimental control and we searched for genes that were increased or decreased more than ten-fold for further study.

The results obtained were then analyzed using the comparative C_T_ method and the miRNA levels were normalized to miRNA-16.

### miRNA qPCR

miRNA samples (2 ng/*μ*l) were reverse transcribed using the TaqMan^®^ microRNA reverse transcription kit (Applied Biosystems; Birchwood Boulevard, Warrington, Cheshire, WA3 7QH). Following manufacturer’s instructions, a master mix was prepared containing 100 mM dNTPs, reverse transcriptase, 10× RT buffer, RNase inhibitor and RNase-free water. The master mix and the TaqMan miRNA 16 (cell extracts only), 933, 144, and 146 5× primer (Applied Biosystems) were added to the miRNA samples and thermocycler conditions included: 30 min at 16°C, 30 min at 42°C, and 5 min at 85°C with a final hold at 4°C.

PrecisionPLUS 2× qPCR MasterMix (Primerdesign Ltd; York House School Lane, Chandler’s Ford, Eastleigh SO53 4DG) and Taqman miRNA 16, 933, 144, and 146a 20× primers (Applied Biosystems) were used for PCR and expression levels were determined using the Stratagene Mx3000P. miR16 was used as the housekeeper gene. Thermal cycling conditions; 10 min at 95°C, 15 s at 95°C and 1 min at 60°C (40 cycles), 15 s at 95°C and 1 min at 60°C and 15 s at 95°C.

Using a spike in control miR from *C. elegans* (mir-39) to calibrate the efficiency of miR extraction from three plasma samples for each subject group, we showed that recovery was 91%, 89%, and 94% for control, AD and AD plus groups respectively and there was no significant interaction between disease and ability to extract miR.

### Statistical analysis

Statistical significance was tested by using ANOVA with Tukey’s post-test, student’s T test or Mann-Whitney U test for non-parametric data using Prism 6 (Graphpad).

## RESULTS

### 27-OHC induces oxidative and inflammatory stress in HMVEC

**Fig. 1.**
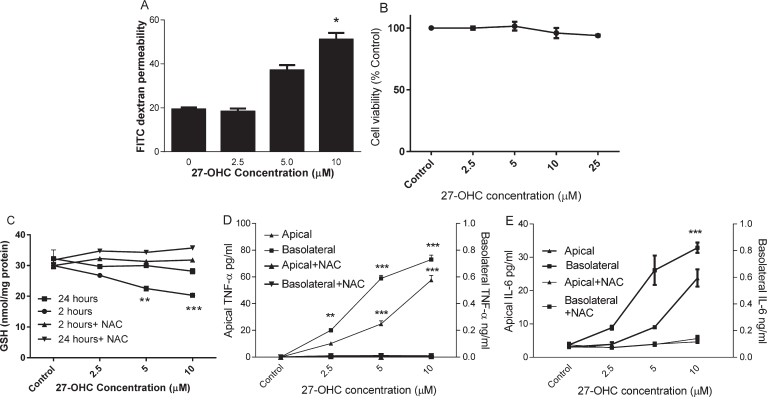
The effect of 27-OHC on endothelial barrier permeability. HMVEC cells were seeded in Transwell inserts for 2 weeks before treating 27-OHC at 2.5, 5 or 10*μ*M for further 24 h with or without 3mM NAC. Barrier integrity (A), viability (B), Intracellular GSH (C; at 2 and 24 h) and secreted TNF-*α* and IL-6 levels (D and E) were measured. ^**^p < 0.01, ^***^p < 0.001, *n* = 3.

HMVEC were cultured on Transwells until day 7 when a tight barrier was formed as described previously [18]. HMVEC monolayers were subsequently exposed to 2.5, 5, or 10*μ*M 27-OHC for up to 24 h. FITC dextran permeability was significantly increased (Fig. 1A) but cell viability was not changed (Fig. 1B) after 24 h incubation with up to 25*μ*M 27-OHC. To understand whether oxidative stress was induced by 27-OHC, we examined intracellular reduced GSH in HMVEC after 2 h and 24 h of oxysterol exposure. 27-OHC (>2.5*μ*M) significantly depleted cellular GSH concentration at 2 h and this was restored to control concentrations after 24 h (Fig. 1C). There was no corresponding increase in GSSG under these conditions (data not shown). The loss of GSH at 2 h could be prevented by co-incubation with 3 mM NAC. After 24 h exposure to 27-OHC, a significant increase in both apical and basolateral secretion of TNF-*α* and IL-6 was observed (Fig. 1D, E). These effects were mitigated by co-incubation with 3 mM N-acetyl cysteine, a precursor for glutathione synthesis. At lower concentrations of 27-OHC, basolateral secretion was affected most and TNF-*α* secretion was more sensitive to 27-OHC (5*μ*M; *p* < 0.01) than IL-6.

### 27-OHC induces miR-933 expression in HMVEC

**Fig. 2.**
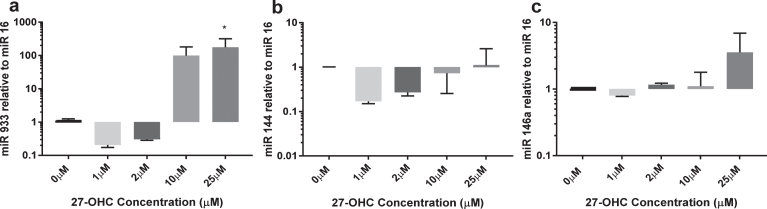
Expression of miR 144, miR 146a, and miR 933 in response to 27-OHC. HMVEC cells (1×10^6^) were treated with 10*μ*M or 25*μ*M 27-OHC for 24 h. Total micro RNA was extracted as described. miR levels were quantified by qRT-PCR relative to miR16. ^*^p < 0.05, *n* = 3.

Using a single microarray discovery experiment, we observed the presence of miR-933 versus an absence in the experimental control, with a *p* < 0.05 and a two-fold higher expression. We selected miR-933 for validation using qRT-PCR. A summary of predicted target genes listed in Table 2 suggests that BDNF is the top target gene for miR-933. In addition, we studied two other miR (miR 144 and miR 146) that are expressed by endothelial cells and are reported to be sensitive to oxidative stress (Fig. 2). miR-933 was observed at very low concentrations in untreated endothelial cells (Ct values >30); however, 25*μ*M 27-OHC elicited more than 100-fold increase in expression of miR-933. miR 144 expression remained unchanged after 24 h exposure to 27-OHC (1–25*μ*M). The expression of miR 146a was increased up to three-fold at the highest concentration of 27-OHC tested (25*μ*M) (Fig. 2C). Low concentrations of 27-OHC (<10*μ*M) had no effect on expression of any of the miR studied.

**Table 2:** Putative targets for miR933 from TargetScan database

Target gene	Gene name
BDNF	brain-derived neurotrophic factor
COL12A1	collagen, type XII, alpha 1
RAP2B	RAP2B, member of RAS oncogene family
KCMF1	potassium channel modulatory factor 1
KPNA1	karyopherin alpha 1 (importin alpha 5)
DAB2IP	DAB2 interacting protein
PEA15	phosphoprotein enriched in astrocytes 15

### 27-OHC and miR in plasma from patients with dementia

To explore whether miR-933 secretion into the circulation was more prevalent in dementia, we examined the plasma from patients with and without AD and vascular dementia. The demographics of the cohorts is shown in Table 1. ApoE4 allele frequency was greater in dementia patients (Table 1). Cognitive function of patients with AD with vascular co-morbidities, AD and healthy control subjects were determined by combination of two well-known cognitive screening brief tests; Mini-Mental Status Examination (MMSE) and clock drawing test. The comparison of the cognitive scores by non-parametric Mann-Whitney analysis shows that both AD plus and AD groups have significant (*p* < 0.0001) cognitive deficits compared to healthy controls.

**Fig. 3.**
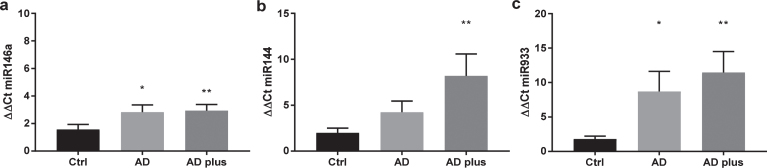
Expression of miR 144, miR 146a, and miR 933 in plasma from older adults. miRNA was extracted using the miRNeasy kit and miR quantitated by qPCR for 10 subjects in each group. The demographic profile of subjects is shown in Table 1.

To explore whether plasma from patients with dementia have increased levels of circulating miR-933, total miR was extracted from the plasma of each patient individually from the three groups, AD, AD-Plus, and control subjects. In common with previous studies, miR 144 and 146a were elevated in plasma from dementia subjects by approximately two orders of magnitude (Fig. 3). In contrast, miR933 was scarcely detectable in healthy subjects (Ct>35) and was found at ten times the order of magnitude in plasma from dementia patients (Fig. 3). AD-plus patients tended to higher levels of circulating miR than AD patients.

**Fig. 4.**
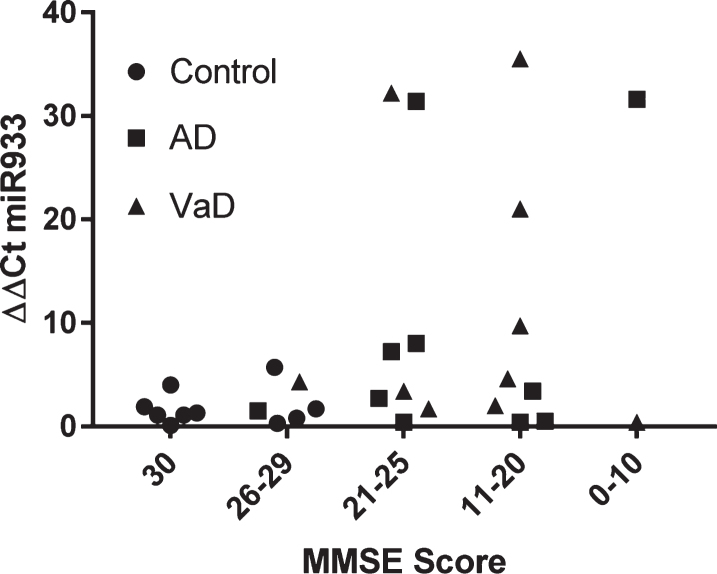
Stratification of miR 933 according to cognitive impairment represented by MMSE scores.

By way of further analysis, we explored whether there was any relationship between cognitive function and plasma miR933. On the population of 30 subjects examined, there was no significant association between MMSE score and plasma miR 933 (Fig. 4).

## DISCUSSION

Previously we have shown that LDL and its lipids can increase the permeability of a HMVEC barrier and have suggested that endothelial cells which line the BBB are likely to be critical mediators of systemic metabolic effects within the brain [16, 18]. We have expanded these studies to examine a specific oxysterol, 27-OHC, which is found at different concentrations in AD patient plasma, cerebrospinal fluid, and brains and has neurotoxic properties [17]. Here we identified for the first time that miR-933 expression is increased in HMVEC by 27-OHC. Others have suggested that miR-933 can interfere with nerve growth factor translation [23] and might therefore be play a role to inhibit BDNF secretion. Using a HMVEC model, we have confirmed that 27-OHC releases discrete inflammatory molecular signatures, comprising of TNF*α* and IL-6, preferentially into the “brain” compartment; this effect is endothelial redox state-dependent. Cytokine-producing endothelial cells also secrete large quantities of EVs [24]. EVs are known to be important cargo vehicles for releasing active molecules, e.g., miR and lipids to distant sites [25]. EVs generated by epithelial cells in the choroid plexus have been observed within the cerebrospinal fluid, supporting the BBB as a point of entry of systemic EVs into the brain [26].

Neuronal EVs are observed with higher concentrations of amyloid and tau in plasma from patients with dementia [27]. Here we have shown that AD patients with vascular complications have higher circulating concentrations of miR-933, miR-146a, and miR-144 than control subjects. We have not explored whether these miR are free or are within EVs. Our report is the first to identify elevated circulating levels of miR-933 in dementia. With enhanced permeability and the potential for directional secretion from endothelial cells into the brain, miR-933 may act as a paracrine inhibitor of neuronal BDNF expression.

Several studies have explored miR144 in relation to dementia; it is regulated by the redox sensitive transcription factor AP1 and it has been shown to inhibit expression of ADAMT-10, which in turn is an inhibitor of A*β* production [28–30]. An increase in miR144 increases A*β* production in neuronal cells.

miR146a, an inhibitor of innate immune signaling, is induced by NF-kB and activates a negative feedback loop inhibiting inflammatory responses [31]. Reduced expression of miR-146a links subclinical inflammation and insulin resistance in type 2 diabetes mellitus. Paradoxically, we show that miR-146a is increased in people with dementia and increased expression of miRNA-146a has been reported in AD transgenic mouse models [32]. miRNA 146a is also increased in brain during systemic inflammation [26]. This may represent an adaptive response that aims to limit inflammation. The increased expression of miR-144 and 146a in plasma from patients with dementia may be a consequence of EV secretion from a range of different cells beyond endothelial cells including inflammatory cells such as monocytes.

Histological studies have shown increased inflammatory cyclooxygenase expression, oxidative stress, and more oxidized lipid deposition in the postmortem brain [33, 34]. The widespread epidemiological association between insulin resistance, obesity, and subclinical inflammation with the loss of cognitive function has been further substantiated recently by changes in the metabolic regulatory miR-29a, miR-335, and miR-125b in the brain [35]. However, most of these studies, including ours has only focused on individual miRNAs, and the sample sizes are too low to conclude diagnostic potential. Therefore, extensive and genome-wide analyses of the miRNAs in the circulating blood of dementia patients are still needed in large-sample studies for disease stratification.

Vascular risk factors appear to influence the future risk of dementia, in particular vascular and mixed-type AD [36] but no clear effects of lipid-lowering or anti-inflammatory interventions are evident in patients with established disease. In a recent study, we have observed that 27-OHC is not modulated by statin intervention in people. This is consistent with an enzymatic synthetic pathway rather than auto-oxidation of cholesterol, hence lipid lowering drugs would not be likely to ameliorate any elevation in 27-OHC seen in dementia [37]. In the present study, we show that after treatment with 27-OHC, there is an associated switch the regulatory miR profile in endothelial cells towards modulating inflammation and inhibiting trophic factor expression.

Here we have investigated 27-OHC as a putative mediator of systemic metabolic effects into the brain compartment in an *in vitro* microvascular endothelial model. Using this approach, we identified both neuromodulatory and inflammatory effects of 27-OHC. We have identified a previously unrecognized target miR as a possible anti-neurotrophic factor and this has led us to investigate and identify the same miR at elevated concentrations in dementia patients. It is beyond the scope of this study to investigate the cause of miR-933 upregulation *in vivo*. In considering whether oxysterols such as 27-OHC may exert any pathophysiologic effect *in vivo*, e.g., on miR-933, it is important to be cognizant of the low concentrations of oxysterols (∼5*μ*M) found *in vivo*, their potential to bind to oxysterol binding proteins and therefore transient nature. miR-933 represents a specific oxysterol-sensitive circulating miR that is found at elevated levels in dementia patient plasma.

Our data highlight that 27-OHC has an important regulatory effect on endothelial microvascular cells and can increase expression of miR-933. Redox-dependent directional secretion of inflammatory cytokines and alterations in neurotrophic factor regulatory miR expression are induced in endothelial cells by 27-OHC in a pattern that is consistent with neurotoxic and inflammatory stress. A miRBase scan revealed that miR-933 may target BDNF and therefore anti-miR933 may be usefully explored for neuroprotective properties, for example in prodromal disease models.
